# Self-Regulation Failure? The Influence Mechanism of Leader Reward Omission on Employee Deviant Behavior

**DOI:** 10.3389/fpsyg.2021.558293

**Published:** 2021-04-07

**Authors:** Tian Wang, Zhoutao Cao, Xi Zhong, Chunhua Chen

**Affiliations:** ^1^College of Economics & Management, Shandong University of Science and Technology, Qingdao, China; ^2^School of Business Administration, South China University of Technology, Guangzhou, China; ^3^National School of Development, Peking University, Beijing, China

**Keywords:** moral disengagement, deviant behavior, leader reward omission, machiavellianism, self-regulation theory

## Abstract

Contingent reinforcement behavior is generally regarded as one of the key elements of being a “good” leader, yet the question of what happens when this behavior is absent has received little attention in past empirical research. Drawing upon self-regulation theory, we develop and test a model that specifies the effects of leader reward omission on employes’ deviant behavior. Using the data of 230 workers from two manufacturing companies located in South China collected across three time points, we find that leader reward omission is positively associated with deviant behavior. Moreover, the indirect effects of leader reward omission on employes’ deviant behavior are mediated by moral disengagement. Our study also reveals that Machiavellianism can aggravate the positive effect of leader reward omission on moral disengagement, and subsequently exacerbate the indirect effect on employes’ deviant behavior. Taken together, our findings reveal the consequences of leader reward omission, and the importance of examining subordinate self-regulation under the lack of positive reinforcement.

## Introduction

Existing literature widely acknowledges the importance of active behavioral reinforcement from supervisors to encourage positive behavior among employes (Podsakoff et al., 2006; Hiller et al., 2011; Behrendt et al., 2017; Hoch et al., 2018; Hu et al., 2021). However, we know little about what happens when active reinforcement is missing. This is known as ‘leader reward omission,’ a term which refers to the absence of positive reinforcement and is characterized by a lack of response from supervisors to good performance by subordinates (Hinkin and Schriesheim, 2015; Holtz and Hu, 2017). Some existing studies have pointed out that non-response to positive outcomes lead to strong negative reactions from employes, such as affective responses, e.g., job dissatisfaction, poor organizational commitment, dissatisfaction with supervisors, turnover intention and burnout) (Hinkin and Schriesheim, 2008b, 2015; Usman et al., 2021), and behavioral responses, e.g., decreases in performance and voice (Qian et al., 2018; Zhang et al., 2020). It is unclear whether leader reward omission increases the possibility of workplace deviance, defined as “voluntary behavior that violates significant organizational norms and, in so doing, threatens the well-being of the organization or its members, or both” (Robinson and Bennett, 1995; Bennett and Robinson, 2000). It is worth noting that the failure of superiors to reinforce proper behavior among subordinates seems to be very common in the workplace (c.f. Aasland et al., 2009; Holtz and Hu, 2017), and may be as important as active leadership behavior, such as transformational leadership (Podsakoff et al., 1990), authentic leadership (Avolio and Gardner, 2005), ethical leadership (Brown and Treviño, 2006), and servant leadership (Hunter et al., 2013). Therefore, it is important to explore whether leader reward omission causes employes to engage in deviant behaviors.

We focus on the process of individual self-regulation impairment to explain the disruptive behavioral outcome (i.e., the extent of subordinates’ deviant behavior) of leader reward omission drawing upon self-regulation theory (Baumeister and Heatherton, 1996). This theory emphasizes that individuals have limited self-regulatory resources to process and deal with uncomfortable reactions caused by physiological processes and environmental pressures (Beal et al., 2005; Koopmann et al., 2015; Mitchell et al., 2019). When the demand for self-regulation exceeds the supply of the resource, the individual loses control in other zones, causing them to experience self-regulation impairment and weakening their ability to restrain improper impulses and harmful counterattacks on targets that are not the source of their original frustration (Baumeister et al., 1998; Vancouver, 2000; Sommovigo et al., 2020). This phenomenon is called displaced aggression (Bushman et al., 2005), and is very common when employes suffer self-regulation impairment (Koopmann et al., 2015; Sommovigo et al., 2020). Thus, self-regulation impairment provides a key perspective to explain subordinates’ deviant behavior as a non-deliberate response to leader reward omission.

Based on self-regulation theory (Baumeister and Heatherton, 1996), we propose an underlying mechanism in which leader reward omission has an indirect effect on deviant behavior via moral disengagement (i.e., the selective suspension of internal self-regulation standards that prevent people from committing inhumane or reprehensible actions (Bandura, 1999)). Specifically, we suggest that employes need to consume self-regulatory resources to deal with the mismatches in their expectations and the reality of supervisors’ rewards, and this process may weaken effective self-control and induce individuals to ignore their own internal moral norms to engage in moral disengagement, allowing deviant behavior to occur.

It would be a mistake to neglect the possible moderating role of individual characteristics when we explore the influence of leader reward omission on individual self-regulation processes based on the self-regulatory theory. Previous studies have pointed out that self-control can be affected by the relative strength of certain individual traits (such as self-protection and competition) (Baumeister and Heatherton, 1996). As such, we introduce Machiavellianism as a moderating variable on the relationship between leader reward omission and moral disengagement. According to Dahling et al. (2008), Machiavellianism is conceptualized as “a tendency to distrust others, a willingness to engage in amoral manipulation, a desire to accumulate status for oneself, and a desire to maintain interpersonal control” (p. 227), which is linked with greater hostility, low morale, and increased work-related stress (Barrick and Mount, 1991; LePine and Van Dyne, 2001). The literature suggests that employes with a high level of Machiavellianism are more concerned with their own interests, and are thus more likely to engage in moral disengagement and negative coping strategies when faced with mismatches in their expectations and the reality of supervisors’ rewards (Christian and Ellis, 2014; Ruiz-Palomino and Linuesa-Langreo, 2018). The key to integrating this construct into the theoretical model is the assumption that self-regulation failure is the result of the interaction of individual difference and environmental cues (Baumeister and Heatherton, 1996).

**FIGURE 1:**
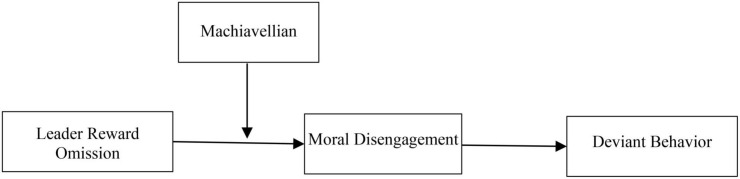
Theoretical model of hypothesized relationships.

In this study, in order to explain the influence of leader reward omission on deviant behavior, we propose and test empirically a conceptual model (see Figure 1) using the data of 230 workers from two manufacturing companies located in South China collected across three time points. This research makes several contributions to the theories in the field of ineffective leadership. First, by exploring the influences of leader reward omission on employes’ deviant behavior, we offer a more comprehensive understanding of the behavioral outcomes of leader reward omission. Second, we uncover the influence mechanism of leader reward omission on workplace deviance by introducing moral disengagement as the mediating variable. Finally, our study further contributes to the literature on leader reward omission by exploring boundary conditions and how Machiavellianism shapes the impacts of leader reward omission on moral disengagement. Overall, this study contributes to our understanding of the consequences and underlying mechanisms of leader reward omission, while also providing recommendations for organizations to improve the clarity of rewards and to offset the negative effects of leader reward omission.

## Theory and Hypotheses

Hinkin and Schriesheim (2015) suggested that social exchange theory is more relevant than other existing theories in understanding the effects of leadership reinforcement omission on employes’ outcomes. Social exchange theory explains social behavior based on the assumption that an individual is a rational actor under the guidance of self-interest (Blau, 1964). The core belief is reciprocity, which is the idea that individuals return positive treatments in a positive way and reciprocate harmful treatment in a vindictive manner (Jacobs, 1970; Cropanzano and Mitchell, 2005). However, there are significant limitations in terms of explaining the effect of leader reward omission on subordinates’ deviant behaviors. That contradicts the prediction of the rule of reciprocity. Specifically, as a member of an organization, an employe relies heavily on organizational resources, such as salaries, inter-personal trust, and information resources, in the process of personal career development (Foa and Foa, 1980). The cost for violating norms or doing harmful to the organization are likely to be greater than the benefits (Thau and Mitchell, 2010). Moreover, the principle of Collectivism in China that advocates employes to primarily be concerned with the objectives and needs of their organizations (Hofstede, 2001), which makes them less likely to engage in deviant behavior, e.g., Wang et al. (2020) suggested that the relationship between job attitudes and workplace deviance is weaker within collectivistic than within individualistic cultures.

In this study, we employ self-regulation theory to explore the relationship between leader reward omission and deviant behavior, and suggest that subordinates’ deviant behavior is more unintentional than purposeful response to leader reward omission in this study. This theory has been proved to be helpful for understanding the individuals’ non-deliberate reaction to interpersonal injustice (Koopmann et al., 2015) and harmful behavior (Mawritz et al., 2017). It emphasizes that self-regulation is the process of using limited self-regulatory resources to inhibit and suppress aggressive impulses and pressures triggered by situations (e.g., unfairness), and control subsequent impulsive behavioral reactions (Baumeister and Heatherton, 1996; Baumeister and Vohs, 2007; Thau and Mitchell, 2010; Mitchell et al., 2019). Based on this theory, we argue that leader reward omission is unfair treatment for subordinates that may aggravate the loss of self-regulatory resources, lead to self-regulation impairment, and ultimately encourage employes to engage in deviant behavior. Moral disengagement specifically depicts an individual’s self-regulation impairment, wherein the individual abandons moral judgment, which is an inappropriate impulse (Bandura, 1999). In addition, Machiavellianism, as a personality trait (Dahling et al., 2008), interacts with leader reward omission (the situational cue) to encourage employes to engage in moral disengagement. When these factors combine, employes are likely to adopt deviant behaviors in response to inappropriate impulses. From the perspective of self-regulation, the conceptual model of this study indicates a comprehensive view of how leader reward omission promotes deviant behavior among employes.

### Leader Reward Omission and Deviant Behavior

The influence of leadership behavior on subordinates is particularly significant in the workplace (Hiller et al., 2011; Hoch et al., 2018). As an agent of an organization, the leader has the responsibility and ability to provide employes with feedback on their performance (Hinkin and Schriesheim, 2015). At the same time, employes tend to seek “evaluation” based on their interactions with their supervisors, speculating on their supervisors’ views and attitudes toward them (Wang et al., 2005). The contingent reinforcement of employe performance by leaders not only involves performance feedback (instrumental influence), but also a timely correction and affirmation in the process of task execution, in this process, superiors also provide “organizational recognition” (emotional influence) by presenting their views on the performance of their subordinates (Hinkin and Schriesheim, 2015). Performance feedback and organizational recognition enable employes to meet their basic needs, enhance their self-awareness and perception of justice, and strengthen their sense of controllability and predictability regarding their work environment (Walumbwa et al., 2008; Tremblay et al., 2013).

Leader reward omission focuses on the lack of positive reinforcement — specifically, when superiors offer no response to good performance from subordinates (Hinkin and Schriesheim, 2008a,b), which not only makes the actual returns from their work inconsistent with their expected returns, but also leaves subordinates unable to obtain performance feedback and organizational recognition. This kind of inequitable treatment then spurs subordinates to perceive a substantial level of uncertainty and insecurity due to the unpredictability of exchange relationships with leaders, exposing subordinates to huge threats and pressure (Mawritz et al., 2017; Ågotnes et al., 2018). The negative experience of being ignored and mistreated by superiors causes subordinates to struggle to handle, explain, and dissolve the reasons and aftereffects of reward omissions (Baumeister et al., 1998). Following a self-regulation failure argument, a series of self-regulation activities would consume a large quantity of self-regulation resources (e.g., vigor and attention), which are also required for moral cognition and considering the risks of retaliation (Bandura et al., 1996; Baumeister and Heatherton, 1996). This means that employes are unable to restrain their desires for deviance due to the exhaustion of their self-regulating resources, and are thus more likely to implement deviant behaviors on targets that are not the source of their original frustration (Baumeister et al., 1998; Vancouver, 2000), which is very common when employes suffer self-regulation impairment (Bushman et al., 2005; Koopmann et al., 2015; Sommovigo et al., 2020). Previous studies have pointed out that self-regulation errors are related to a variety of unhealthy interpersonal interactions (Stucke and Baumeister, 2006; Hagger et al., 2010). Therefore, this study argues that self-regulation obstacles prompt employes to unconsciously and unintentionally implement deviant behaviors. The following hypothesis is proposed:

Hypothesis 1: There is a positive correlation between leader reward omission and employes’ deviant behavior.

### Mediating Role of Moral Disengagement

The idea of moral disengagement was originally proposed by Bandura (1990, 1991a). It represents self-regulatory breakdowns and is used to explain how individuals exercise cognitive control of their attention and behavior by engaging in the process of self-regulation (Bandura, 1990, 1991a). An individual is inclined to inhibit actions contrary to their ethical standards, as long as their self-regulation mechanism is properly activated and operated (Bandura, 1991b; Bandura et al., 1996). Some existing researches suggest that the negative experience for employes might increase the possibility of self-regulation failure, then induce individuals to ignore their own internal moral norms to engage in unethical cognitive judgments and impulsive, unethical behaviors (Christian and Ellis, 2014). For example, Hystad et al. (2014) and Loi et al. (2015) found that moral disengagement mediated the relationship between organizational injustice and unethical behaviors.

Leader reward omission is a negative experience for employes. It refers to a lack of leadership feedback and evaluation regarding employe performance, prompting employes to be confused about the alignment between their work behavior and organizational goals, leading to role ambiguity or confusion about job expectations (Rizzo et al., 1970). Receiving no explicit rewards for great performance is contrary to the existing implicit and explicit behavioral norms within organizations. This inconsistency between expectation and reality thus reduces employes’ satisfaction with their supervisors, harming their self-esteem and bringing them under psychological pressure (Thau and Mitchell, 2010). Negative experiences cause subordinates to consume a considerable amount of self-regulating resources, meaning they can no longer invest enough strength in the process of moral awareness and decision-making. This leads to self-regulation failure, causing employes to lose their self-discipline and violate their own moral standards (Baumeister et al., 2006; Yam et al., 2014), and engage in unethical cognitive judgments and impulse (Christian and Ellis, 2014). This impulse is hard to restrain, further allowing individuals to consciously engage in unethical behaviors as a coping strategy while maintaining a positive assessment of themselves, assuming that these behaviors will benefit either themselves or the organization (Newman et al., 2019). In sum, we propose that leader reward omission may improve moral disengagement.

Acting in an immoral or deviant manner depends on an individual’s cognitive ability to control their emotions and behavior (Christian and Ellis, 2011). Similarly, Baumeister and his colleagues proposed that when an individual fails to self-regulate their behavior, it will instead be largely determined by improper motivations and tendencies, with the individual then engaging in unethical behaviors without restrictions (e.g., Baumeister et al., 2006; Baumeister and Vohs, 2007), such as deception, lying, and theft (Fida et al., 2015). Therefore, we believe that moral disengagement is an important precursor to deviant behavior, a claim that is supported by empirical evidence (Moore et al., 2012; Dang et al., 2017). Zheng et al. (2019) uncovered the mediating effect of moral disengagement on the relationship between individual creativity and workplace deviant behavior by drawing on moral self-regulation theory. Moral disengagement provides an acceptable way for individuals to consider their destructive behavior, as well as its unfavorable consequences, making it easier to manage cognitively (Bandura, 1991a; Bandura et al., 1996). Overall, moral disengagement is an important mechanism for understanding the formation of deviant behaviors in the workplace (Newman et al., 2019), presenting such behaviors from leading to feelings of guilt (Fida et al., 2015). Based on the above analysis, the following hypothesis is proposed:

Hypothesis 2: Moral disengagement mediates the positive relationship between leader reward omission and deviant behavior.

### Moderating Role of Machiavellianism

Self-regulation theory claims that the main goal of individual self-regulation is to maintain the individual’s behavior in line with social norms. It is worth noting that there is only one standard for appropriate or expected behavior in many cases, but the actual response patterns vary based on individual circumstances (Baumeister et al., 2006). Based on this, the consumption process of individual self-regulation resources is also influenced by an individual’s personality, including aspects such as their tendency toward Machiavellianism, which emphasizes individuals’ pursuit of maximizing their self-interest. Existing research points out that having a dark personality has strong explanatory power regarding why individuals engage in unethical behaviors (Christian and Ellis, 2014). However, few studies have explored the influence of Machiavellianism on the process of self-regulatory resource consumption. In order to support the existing literature, Machiavellianism is introduced as a moderating variable to better understand the boundary conditions of leader reward omission on moral disengagement.

Christie and Geis (1970) suggest the concept of Machiavellianism as a social behavioral strategy that involves manipulating others for individual interests (Dahling et al., 2008). Individuals with Machiavellian tendencies often have the following characteristics: a lack of empathy; low levels of emotion; a focus on pursuing their own goals at the expense of others; and abnormal moral values (Moore et al., 2012). It is believed that individuals with Machiavellianism do not lack moral standards (Schepers, 2003), but tend to ignore moral norms or engage in moral disengagement (Dahling et al., 2008). In accordance with self-regulation theory, this study argues that Machiavellianism focuses on selfishness and self-interest, which have a significant influence on employes’ self-regulation processes. Specifically, compared with low-level Machiavellians, people with high-level Machiavellians tend to emphasize their own personal interests, and it is more difficult for them to accept their superiors’ failure to response to their ‘excellent performances.’ This inequitable treatment causes them to experience more negative emotions, such as uncertainty and unfairness. As such, they cannot help but spend more self-regulatory resources on understanding and explaining the reasons for the unfair treatment. Self-regulation theory argues that self-regulation resources are limited, with individuals losing the ability to self-control against aggressive impulses when these resources are exhausted (Baumeister and Vohs, 2007; Yam et al., 2014). In such cases, moral disengagement is more likely to occur. Moreover, Machiavellians, who do not lack moral standards (Schepers, 2003), tend to ignore moral norms or engage in moral disengagement (Dahling et al., 2008; Gabi et al., 2019). Therefore, we suggest that subordinates with high-level Machiavellians are more inclined to respond to an abuser in an unethical way in the face of the lack of reward. Based on the above analysis, this study suggests that when employes experience leader reward omission, high-level Machiavellian individuals have to spend more self-regulation resources, are more likely to fail in their attempts to self-regulate, and are more likely to conduct in moral disengagement, when compared to low-level Machiavellians. Based on above synthesis of the current literature, the following hypothesis is proposed:

Hypothesis 3: Machiavellianism moderates the relationship between leader reward omission and moral disengagement. When Machiavellianism is higher, leader reward omission has a more significant effect on employes’ moral disengagement.

Given the above, we describe the process of individual self-regulation under the interaction of conditional influence (leader reward omission) and individual influence (Machiavellianism) by employing self-regulation theory. Within this theory, individuals cannot control undesirable impulses (moral disengagement) and subsequent behavioral responses (deviant behavior) when they run out of self-regulatory resources and experience self-regulation impairment. We suggest that moral disengagement mediates the positive relationship between leader reward omission and deviant behavior (Hypothesis 2). In addition, Machiavellianism strengthens the effect of leader reward omission on employes’ moral disengagement (Hypothesis 3). A combination of individual and environmental influences would consume more self-regulatory resources than a single factor and increase the likelihood of self-regulation failure, as well as strengthening the impulse toward moral disengagement and further increasing the subsequent behavioral response (deviant behavior). Combining hypotheses 2 and 3, this study proposes a moderated mediation model. The degree of moral disengagement is influenced by the level of individual Machiavellianism. Individuals with high Machiavellianism are more likely to experience an adverse response to leader reward omission, making it easier to activate the mechanism of moral disengagement and thus engage in deviant behavior. On the other hand, for individuals with low Machiavellianism, these effects might be weaker. The following hypothesis of moderated mediation is proposed:

Hypothesis 4: Machiavellianism moderates the mediating effect of moral disengagement between leader reward omission and deviant behavior. When Machiavellianism is higher, the mediating effect is greater than when Machiavellianism is lower.

## Method

### Participants and Procedure

We conducted a longitudinal study among two manufacturing companies located in South China. Through the gatekeeping of the companies’ human resources managers, 400 participants voluntarily participated in the survey, including participants from different departments and fields, such as product design, engineering, quality control, marketing, and human resources. We asked all volunteers to sign their informed consent before taking part in the study, and informed participants that they had the option to exit at any point. Data were collected in three periods in order to minimize common method bias (Podsakoff et al., 2003). In the first survey (Time 1), we told the participants the purpose of the survey and asked them to report their perceptions of leader reward omission, their levels of Machiavellianism, and basic demographic information, including age, education and sex. After two months (Time 2), the participants evaluated their levels of moral disengagement and creative self-efficacy. Two months after the second questionnaire (Time 3), the participants completed the measures for deviant behavior. Consistent with prior studies, we used a self-reported deviance measure. This is because many deviant behaviors are difficult to observe (Fox et al., 2001). We promised participants that their responses would be kept confidential in order to limit the possibility of evaluation apprehension and social desirability bias.

At Time 1, we received 356 completed questionnaires, yielding a response rate of 89%. At Time 2, questionnaires were distributed to the same 356 participants who completed the Time 1 survey. A total of 302 participants completed the Time 2 survey, yielding a response rate of 84.83%. For Time 3, 257 questionnaires were completed, yielding a response rate of 85.1%. After deleting invalid questionnaires, 230 valid questionnaires were generated, with an effective response rate of 57.5%. In the final sample (*N* = 230), the number of male (51.7%) and female (48.3%) participants was similar; 91.7% of participants were under 35 years old; and 84.8% held a bachelor’s degree.

### Measures

We conducted all the surveys in Mandarin Chinese, and translated the English scales into Mandarin Chinese following Brislin’s (1970) translation–back translation procedure. We adopted this procedure to maximize the equivalence between the translated scale and the original scale in terms of content and meaning. Considering the deviation of the homology method, and the influence of social approval and concealment of research variables on the hypothesis validation analysis, we emphasized the anonymity of the questionnaire to the employes before issuing it.

Leader reward omission (Time 1) was measured using the scale developed by Hinkin and Schriesheim (2008b). An example item was “I have been working well, but I have not been praised by the leaders.” Participants rated their supervisors’ reward omission on a 5-point Likert-type scale ranging from 1 (never) to 5 (always).

Machiavellianism (Time 1). We used a 16-item measure developed by Dahling et al. (2008) to assess the level of Machiavellianism. An example item was “If the probability of being caught is very low, I will cheat.” We measured all the items on a 5-point Likert-type scale ranging from 1 (strongly disagree) to 5 (strongly agree).

Moral disengagement (Time 2) We used an 8-item measure developed by Moore et al. (2012) to assess the level of moral disengagement. An example item was “it is not important to attribute other people’s ideas to oneself.” We measured all the items on a 5-point Likert-type scale ranging from 1 (strongly disagree) to 5 (strongly agree).

We measured deviant behavior (Time 3) using nineteen items adapted from Bennett and Robinson (2000). Specifically, we replaced the “racial” with “national” for the item “Made an ethnic, religious, or racial remark at work.” In the Chinese business context, the nation is more suitable than race. An example item was “I forged data to get more money than I actually spent on business expenses.” Participants reported their deviant behavior on a 5-point Likert-type scale ranging from 1 (never) to 5 (always).

We measured creative self-efficacy (Time 2) using three items developed by Tierney and Farmer (2002). An example item was “I have confidence in my ability to solve problems creatively.” We measured all the items on a 5-point Likert-type scale ranging from 1 (strongly disagree) to 5 (strongly agree).

## Results

### Preliminary Analysis

Since that we measured the variables using self-reported answers in this study, we employed two methods with a marker variable to test for possible common method bias (CMB). Creative self-efficacy served as the marker variable (Tierney and Farmer, 2002, Cronbach’s α: 0.907). Correlations between creative self-efficacy and leader reward omission, deviant behavior, moral disengagement, Machiavellianism were −0.020, −0.020, −0.082, and −0.018, respectively. The negligible correlation indicated that creative self-efficacy was an appropriate marker variable. Firstly, the correlational marker technique (Lindell and Whitney, 2001) was used in this study. The original correlation coefficient R_u_ between variables was converted by using the formula R_A_ = R_u_ - R_M_/(1-R_M_)^2^ to obtain the correlation coefficient R_A_ which could remove the influence of CMV. The results are shown in Table 1. The lower triangle included the original correlation coefficients, and the upper triangle included the converted coefficients. Obviously, the correlation coefficients among the main variables remained significant. In conclusion, the common method bias in this study is not serious. Secondly, the CFA marker technique (Williams et al., 2010; Podsakoff et al., 2012) was employed in this study. The results of the chi-square difference test showed that adding fixed equal factor load did not significantly improve the baseline model where the marker variable was orthogonal to the main variable items (Δχ2 = 8.099, *df* = 4) (see Williams et al., 2010). In conclusion, although there was inevitable common method bias in this study, it did not have a significant impact on the research results.

**TABLE 1:** Descriptive statistics and correlations of variables.

**Variables**	**1**	**2**	**3**	**4**
1. Leader Reward Omission(T1)	**(0.928)**	0.222**	0.426**	0.411**
2. Deviant Behavior(T3)	0.205**	**(0.925)**	0.467**	0.321**
3. Moral Disengagement(T2)	0.409**	0.450**	**(0.875)**	0.507**
4. Machiavellianism(T1)	0.394**	0.304**	0.490**	**(0.902)**
Mean	2.493	1.887	2.253	2.833
SD	0.970	0.700	0.763	0.758

N = 230, *p < 0.05, **p < 0.01. SD = standard deviation. Bolded values in Cronbach’s alpha coefficients are shown on the main diagonal.

To ensure the discriminant validity of the constructs, we performed a confirmatory factor analysis. In comparison (Table 2), the fitness of the four-factor model to data was significantly better than that of other models (χ2/*df* = 1.978, RMSEA = 0.065, CFI = 0.900, IFI = 0.893, SRMR = 0.084). The Z-score of skewness and kurtosis for all variables (Moral Disengagement, Deviant Behavior, Leader Reward Omission, Machiavellianism) was within ± 1.96, verifying that they all correspond to normal distribution.

**TABLE 2:** Summary of model fit indexes.

**Structure**	**χ*^2^***	***df***	**χ*^2^/df***	***RMSEA***	***CFI***	***TLI***	***SRMR***
Four-factor Model (LRO, MD,M,DB)	2359.520	1193	1.978	0.065	0.900	0.893	0.084
Three-factor Model (LRO, MD + M,DB)	2610.735	1197	2.181	0.072	0.878	0.871	0.093
Two-factor Model (LRO, MD + M + DB)	3212.761	1201	2.675	0.086	0.827	0.816	0.108
One-factor Model (LRO + MD + M + DB)	3994.108	1203	3.320	0.106	0.760	0.746	0.127

*N = 230. LRO = Leader Reward Omission; MD = Moral Disengagement; M = Machiavellianism; DB = Deviant Behavior, “+” means to combine.*

The mean, standard deviation, reliabilities, and correlations of the variables are presented in Table 1. Leader reward omission was positively correlated with employes’ deviant behavior (r = 0.205, *P* < 0.01), as well as being positively correlated with moral disengagement (r = 0.409, *P* < 0.01). Moral disengagement was also positively correlated with employes’ deviant behavior (r = 0.450, *P* < 0.01).

### Hypotheses Testing

The conceptual model was tested using hierarchical regression analysis. The variance inflation factor (VIF) of all variables included in the regression model were equal to or lower than 2. This was well below the limited threshold of 10, suggesting that the multi-collinearity problem in this study was well controlled. The results of the regression analysis are presented in Table 3. Leader reward omission was positively correlated with employes’ deviant behavior (M1, β = 0.205, *p* < 0.01). Thus, Hypothesis 1 was supported.

**TABLE 3:** Regression results for model predicting deviant behavior.

**Variables**	**M1**	**M2**	**M3**	**M4**	**M5**
Leader Reward Omission(T1)	0.205**	0.025	0.409**	0.256**	0.228**
Moral Disengagement(T2)		0.440**			
Machiavellianism(T1)				0.389**	0.414**
Leader Reward Omission* Machiavellianism				0.246**	
Total *R*^2^	0.042	0.203	0.167	0.295	0.355
Δ *R*^2^	0.038	0.196	0.164	0.289	0.347
*F*	9.992**	28.955**	45.855**	47.577**	41.482**

N = 230, *p < 0.05, **p < 0.01. M for model. Standardized regression coefficients reported.

To verify the mediating effect of moral disengagement on the relationship between leader reward omission and employes’ deviant behavior, we used the PROCESS macro program (Model 4) for SPSS (Hayes, 2013) and bootstrapped 5,000 samples to test the model for mediation. As shown in Table 4, the mediating model fits well. Significant indirect effects of leader reward omission (indirect effect = 0.130, 95% CI = [0.070, 0.210]) on deviant behavior via moral disengagement were found. Thus, Hypothesis 2 was strongly supported in this study.

**TABLE 4:** Results of bootstrapping tests with 95% confidence intervals (CI): the mediating roles of moral disengagement between leader reward omission and deviant behavior.

**Predictor**		**Effect**	**SE**	***t***	***p***	***LLCI***	***ULCI***
Leader Reward Omission	Total effect	0.148	0.047	3.161	0.002	0.056	0.240
	Direct effect	0.018	0.047	0.382	0.703	–0.074	0.110

		**Effect**	***Boot S.E.***			***BootLLCI***	***BootULCI***

	Indirect effect	0.130	0.035			0.070	0.210

*N = 230. Bootstrap sample size = 5,000. SE = standard error. LLCI = lower level of confidence interval. ULCI = upper level of confidence interval. Boot S.E. = bootstrap standard error. BootLLCI = bootstrap lower level of confidence interval. BootULCI = bootstrap upper level of confidence interval.*

**FIGURE 2:**
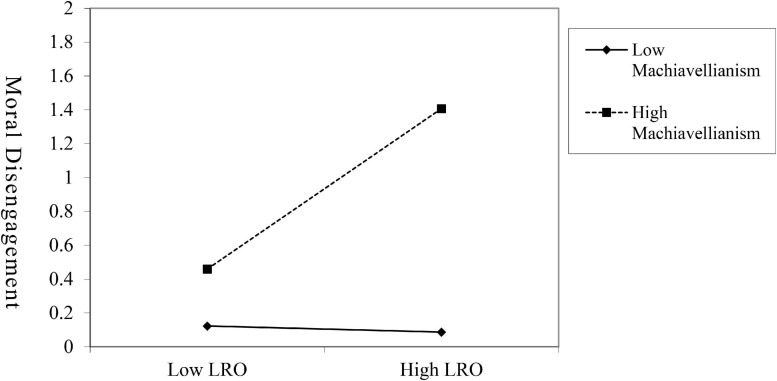
Interaction of leader reward omission and machiavellianism predicting moral disengagement. *N* = 230, LRO = leader reward omission.

Regarding the moderating effect of Machiavellianism proposed in Hypothesis 3 and presented in Table 3, the interaction term (Leader Reward Omission after mean centering ^∗^ Machiavellianism after mean centering) was significantly associated with moral disengagement (M5, β = 0.246, *p* < 0.01). The mean value, and the values plus/minus one SD from the mean of the quantitative moderators, were used as the benchmark to differentiate the high/medium/low groups. Simple slope tests (Figure 2) indicated that the positive relationship between leader reward omission and moral disengagement was significant for participants with high levels of Machiavellianism (simple slope = 0.37, SE = 0.08, t = 4.52, *P* < 0.01) and with medium levels of Machiavellianism (simple slope = 0.18, SE = 0.05, t = 3.32, *P* < 0.01). However, the positive relationship was no longer significant for participants with low levels of Machiavellianism (simple slope = −0.01, SE = 0.07, t = −0.16, *P* = 1.127). It can be seen that leader reward omission has a more significant positive impact on moral disengagement only in the case of high levels and medium levels of Machiavellianism. Thus, Hypothesis 3 was supported.

Finally, this study examined a first-stage moderated mediation model (Edwards and Lambert, 2007) in which the moderating variable (Machiavellianism) was hypothesized to operate on the first stage of the indirect relationship (i.e., between leader reward omission and moral disengagement). This was assessed using the PROCESS macro program (Model 7) for SPSS (Hayes, 2013) with 95% bias corrected and accelerated bootstrapped confidence intervals (CI) based on 5,000 samples. We can see from Table 5 that the indirect effects of leader reward omission on employes’ deviant behavior through moral disengagement was significant for individuals with high levels of Machiavellianism (b = 0.146, 95%IC = [0.075, 0.235]) but not for individuals with low levels of Machiavellianism (b = −0.001, 95%IC = [−0.051, 0.060]). Thus, Hypothesis 4 was supported.

**TABLE 5:** Results of bootstrapping tests with 95% confidence intervals (CI): the moderated mediation roles of machiavellianism between leader reward omission and deviant behavior.

**Predicator**	**Conditional indirect effect**					**Moderated mediation**			
		
	**Machiavellianism**	**Effect**	**Boot SE**	**BootLLCI**	**BootULCI**	**INDEX**	**SE(Boot)**	**BootLLCI**	**BootULCI**
Leader reward omission	–0.729	–0.001	0.029	–0.051	0.060	0.101	0.032	0.044	0.169
	0.000	0.072	0.026	0.030	0.131				
	0.729	0.146	0.040	0.075	0.235				

*N = 230. Bootstrap sample size = 5,000. Boot SE = bootstrap standard error. BootLLCI = bootstrap lower level of confidence interval. BootULCI = bootstrap upper level of confidence interval. SE(Boot) = bootstrap standard error.*

### Robustness Analysis

In order to make the assumptions close enough to reality, this study employed the supplementary variables (gender, age, and educational background) to run a robustness test. Previous research has noted that these characteristics affected employe workplace deviance (Ferris et al., 2009). More specifically, there were differences between men and women in ethical sensitivity and orientation (Ambrose and Schminke, 1999). For example, Khazanchi (1995) and Gonzalez-Mulé et al. (2013) proposed that male participants tended to engage in more deviant behavior. The other characteristics (age and education) also influenced employes’ ethical reasoning and moral behaviors (Loe et al., 2000): people with higher levels of education tended to have higher levels of moral judgment (Rest and Thoma, 1985), and the relationship between age and ethical decision-making was positive. We chose the categories (male; under the of age 26; master’s degree or above) as the reference group, and transformed other categories into dummy variables, after which we put them into regression models. As the results of the robustness analysis showed, the coefficients of the regression model with supplementary variables (gender, age, educational background) were consistent with the results of regression model without supplementary variables (Tables 3–5), and further ensured the robustness of the empirical results^1^.

## Discussion

In addition to the potential resistance [e.g., job satisfaction, organizational commitment, satisfaction with the leader, and turnover intention (Hinkin and Schriesheim, 2008b, 2015)] mentioned in existing literature as an outcome of leader reward omission, we know nothing about whether leader reward omission increases the possibility of workplace deviance. In order to explore this issue, this study developed a moderated mediation model based on self-regulation theory. The findings revealed that leader reward omission was positively associated with subordinates’ deviant behavior, and moral disengagement played a mediating role in the relationship between them. Machiavellianism, as an individual difference, strengthens the positive effects of leader reward omission on moral disengagement.

### Theoretical Implications

Some key contributions of the present study are worth highlighting. First, the current study has explored the impact of leader reward omission on employes’ deviant behavior by examining the process of individual self-regulation based on self-regulation theory, and has expanded the understanding of leadership effectiveness by yielding insights into the impact of leaders’ non-reactions to subordinates’ performance. Specifically, our research reveals that deviant behavior results from the loss of self-control, and that employes engage in deviant behavior only because they do not have enough self-regulatory resources to inhibit the impulse of engaging in deviant behavior. Our research shows that even relatively ‘good’ employes may engage in deviant behaviors in the absence of leadership rewards, because leader reward omission wastes employes’ self- regulatory resources. Previous studies often applied the principle of reciprocity from social exchange theory to parsimoniously explain the relationship between leadership behavior and employe behavior; for instance, “to fight back against unfavorable treatment with negative treatment.” However, it is generally known that employes are largely dependent on their organizations and supervisors in terms of valuable resource acquisition and career development. Furthermore, in a Chinese context, the dependence of employes makes them unlikely to engage in deviant behavior. It can be seen that the social exchange theory could not explain why deviant behavior is generally considered to be socially unacceptable, yet non-etheless occurs. Self-regulation theory provides a reasonable explanation for this phenomenon. Employes must expend large quantities of self-regulatory resources to understand and deal with the negative emotion emerging from the absence of supervisors’ rewards, causing them to have almost no ability to suppress the displaced aggression. Collectively, research into deviant behavior which includes self-regulation frameworks deepens our understanding of this confusing workplace phenomenon.

Second, this study uncovers the underlying mechanism of the impact of leader reward omission on subordinates’ deviant behavior by introducing moral disengagement into the model as the mediating variable. It reveals that moral detachment is a powerful explanation for the deviant behavior resulting from leader reward omission. Most existing studies have only explored the intermediary mechanism of the effect of leadership on employes’ deviance behaviors by drawing on social exchange theory and the principle of reciprocity (Podsakoff et al., 2006; Mayer et al., 2012) without considering the impact of the experience of frustration and anger perceived by employes in their interactions with leaders from a recipient-centric perspective. Based on self-regulation theory, this study finds that this “non-response” of leaders means that employes are treated with indifference and cannot obtain the recognition and rewards they feel that they deserve. In turn, this causes employes to experience psychological exhaustion and lose self-control (Baumeister and Heatherton, 1996). Specifically, the abused employes need to consume a large amount of self-regulating resources to understand and digest the reasons for their victimization. These activities deplete their necessary self-regulation resources and result in self-regulation impairment, inducing them to ignore their own internal moral norms, form unethical cognitive judgments, and conduct unethical behavior impulsively (Bandura, 1991a,b; Bandura et al., 1996; Zhong, 2011). The findings of this study are important in that they provide empirical evidence that self-regulation impairment serves as a stronger theoretical explanation for our proposed theoretical model than social exchange, which suggests that subordinates’ deviance is more unintentional than purposeful. Therefore, this study reasonably enriches research into leader reward omission by explaining the underlying mechanism for how it promotes employes’ deviant behavior from the perspective of self-regulation.

Third, this study demonstrates that Machiavellianism is an important individual difference that plays a significant role in the positive relationship between leader reward omission and deviant behavior. The first-stage moderated mediation model suggests that Machiavellianism positively moderates the mediating effect of moral disengagement on the relationship between leader reward omission and employe deviant behavior. However, individuals with different degrees of Machiavellianism may take different actions to respond to their sense of ill-treatment due to their differentiated understandings and interpretations of their interactions with leaders (Baumeister et al., 2006). Specifically, employes with a higher level of Machiavellianism experience a stronger sense of injustice when superiors do not reward their great performance. It requires more self-regulation resources for them to digest this rejection, leaving them more likely to both experience self-regulation obstacles and engage in moral disengagement. In addition, previous empirical evidence also suggests that the ability to control the self and self-regulation impairment is negatively associated with the intention to engage in moral disengagement and deviant behaviors (Marcus and Schuler, 2004; Baumeister et al., 2005). Overall, this study provides further insight into the boundary conditions of the effect of leader reward omission on moral disengagement by explaining the influence of Machiavellianism in the process of self-regulation based on self-regulation theory.

### Practical Implications

This research has several implications for management practices. First, the study provides a possible explanation for why employes engage in deviant behavior. Leader reward omission is a very important trigger factor, which may lead to employes’ self-regulation failure, thus prompting employes to impulsively engage in deviant behavior. In view of the negative impact of leader reward omission on subordinates and on organizational performance, leaders should reward employes for good work in an active and timely fashion. Specifically, organizations should encourage more communication between supervisors and subordinates. Full and timely communication could help supervisors to understand the work performance of subordinates, so that they can reward excellent performance in time. In addition, organizations should encourage supervisors to provide feedback to their subordinates in a respectful and constructive way and to give full play to subordinates’ abilities, so as to reduce negative emotions and behavioral reactions to unfair treatment. Second, the influence of leader reward omission on deviant behavior can be observed based on the increasingly strong impulse of moral disengagement. Based on this, one option is to test the moral disengagement of candidates in the selection process and weigh the score in the final recruitment decision based on the turnover rate of a given industry. Alternatively, organizations could actively carry out employe moral education, organize feedback seminars or private talks, and establish employes’ moral standards and codes of conduct to prevent employes from experiencing moral disengagement. Finally, the results show that employes with greater Machiavellian tendencies are more likely to experience moral disengagement when leader reward behavior is absent. Therefore, in the process of recruiting new employes, employers should pay more attention to the personalities of employes and exclude potential employes with higher levels of Machiavellianism. In addition, supervisors should be particularly sensitive to employes with a high level of Machiavellianism to prevent them from violating the legitimate and ethical objectives of the organization and engaging in conduct harmful to the organization.

### Limitations and Future Research Directions

There are several limitations that need to be addressed in future research. First, for theoretical reasons, we examined the moderating effects of Machiavellianism, capturing the influence of employes’ individual differences on the self-regulation process. In addition to this factor, future research could take into account organizational contexts (e.g., organizational climate, HR practices, etc.) and analyze the boundary conditions of other personal characteristics, such as mindfulness (Montani et al., 2019) and moral identity (Zheng et al., 2019). Second, although the three-wave design is useful in reducing common method effects, the design is not sufficiently robust to test the cause-and-effect relationship with certainty. It is worth noting that it takes a long time to complete the cognitive transformation. Therefore, more extensive sampling methods in a longitudinal research design could be used in future research to obtain a more accurate understanding of cognitive transformation over time. Third, the sample for this study consisted of two companies in South China. Considering the obvious regional cultural characteristics of China as a country, further research is needed to clarify whether the results of this research can be generalized to other organizations in different countries, as cultural values have been found to influence employes’ reactions to unfair treatments, meaning that individuals from different countries may react differently to the same unfair situation (e.g., Shao and Skarlicki, 2014; Sommovigo et al., 2020). Finally, self-regulation theory was used to identify the mediating effect of moral disengagement on the relationship between leader reward omission and employes’ deviant behavior. However, interpersonal interaction is a very complex behavior and is an indispensable part of daily work. This means that the effects of leader reward omission on employes’ deviant behavior may be influenced by factors other than uncertainty management, such as organizational identity. Future research should explore the intermediary mechanism from other theoretical perspectives.

## Conclusion

This study provides further insight into the operating mechanism of leader reward omission based on the self-regulation theory. Specifically, it finds that leader reward omission is a very important trigger factor which may lead to employes’ self-regulation failure, thus prompting employes to impulsively engage in deviant behavior. Moreover, the influence of leader reward omission on deviant behavior can be seen from the increasingly strong impulse of moral disengagement. Finally, it finds Machiavellianism to be an important personal characteristic that influences the strength of the association between leader reward omission and moral disengagement. This research can help organizations to identify employes who are particularly at risk of moral disengagement, and to take steps designed to prevent the negative effects of leader reward omission.

## Data Availability Statement

The raw data supporting the conclusions of this article will be made available by the authors, without undue reservation.

## Ethics Statement

Ethical approval was not provided for this study on human participants because according to institutional guidelines and relevant laws and regulations, this study does not require ethical approval because it did not involve any tests on people or animals or unethical behavior. The participants provided their written informed consent to participate in this study.

## Author Contributions

TW was mainly responsible for writing the manuscript and analyzing the data. ZC and CC were in charge of revising and improving the manuscript. XZ was responsible for analyzing the data. All authors contributed to the article and approved the submitted version.

## Conflict of Interest

The authors declare that the research was conducted in the absence of any commercial or financial relationships that could be construed as a potential conflict of interest.

## Footnotes

1 The results of robustness analysis can be found in the online appendix.
