# Supplementary material for: Self-Regulation Failure? The Influence Mechanism of Leader Reward Omission on Employee Deviant Behavior
Source: Front Psychol. 2021 Apr 7;12:558293. doi: 10.3389/fpsyg.2021.558293 (PMC8058438; doi:10.3389/fpsyg.2021.558293)
Supplement: Supplementary file 1 [file Data_Sheet_1.pdf]

The result of robustness analysis

In order to make the assumptions close enough to reality, this study employed the supplementary variables (gender, age, and educational background) to run a robustness test. Previous research has noted that these characteristics affected employee workplace deviance (Ferris et al., 2009). More specifically, there were differences between men and women in ethical sensitivity and orientation (Ambrose and Schminke, 1999). For example, Gonzalez (2013) and Khazanchi (1995) proposed that male participants tended to engage in more deviant behavior. The other characteristics (age and education) also influenced employees' ethical reasoning and moral behaviors (Loe et al., 2000): people with higher levels of education tended to have higher levels of moral judgment (Rest et al.,1985), and the relationship between age and ethical decision-making was positive. We chose the categories (male; under the of age 26; master's degree or above) as the reference group, and transformed other categories into dummy variables, after which we put them into regression models. As the results of the robustness analysis showed (Table 6,7,8 and 9, Figure 3), the coefficients of the regression model with supplementary variables (gender, age, educational background) were consistent with the results of regression model without supplementary variables, and further ensured the robustness of the empirical results.

Supplementary Table 1

Descriptive Statistics and Correlations of Variables (N=230) (Robust test)

Variables	1	2	3	4	5	6	7	8	9
1.Gender（0= male）									
2.Age (26-35)	-0.109								
3.Age (>35)	-0.037	-0.351**							
4.Education(College degree or below)	-0.167**	-0.177*	0.181**						
5. Education (Bachelor's Degree)	0.099	0.104	-0.127	-0.548**					
6. Leader Reward Omission(T1)	-0.013	-0.177**	-0.143*	0.030	-0.236**	**(0.928)**			
7.Deviant Behavior(T3)	-0.250**	0.108	-0.077	0.332**	-0.323**	0.205**	**(0.925)**		
8.Moral Disengagement(T2)	-0.119	-0.094	0.058	0.187**	-0.247**	0.409**	0.450**	**(0.875)**	
9.Machiavellism(T1)	-0.265**	-0.100	0.274**	0.113	-0.213**	0.394**	0.304**	0.490**	**(0.902)**
Mean	0.483	0.578	0.083	0.152	0.626	2.493	1.887	2.253	2.833
SD	0.501	0.495	0.276	0.360	.0485	0.970	0.700	0.763	0.758

*p<.05. **p<.01. Cronbach's alpha coefficients are shown on the main diagonal.

Supplementary Table 2

Regression Results for Model Predicting Deviant Behavior(Robust test)

Variables	M1	M2	M3	M5	M6	M7	M8
Gender (0= male)	-0.184**	-0.179**	-0.147**	-0.100	-0.090	0.009	-0.013
Age (26-35)	0.110	0.141*	0.148*	-0.078	-0.020	-0.024	0.025
Age (>35)	-0.112	-0.129*	-0.115	-0.007	-0.039	-0.125*	-0.162**
Education (College degree or below)	0.229**	0.267**	0.225**	0.045	0.117	0.134	0.122
Education (Bachelor's Degree)	-0.205**	-0.142	-0.112	-0.205**	-0.086	-0.036	-0.016
Leader Reward Omission(T1)		0.205**	0.067		0.386**	0.252**	0.237**
Moral Disengagement(T2)			0.357**				
Machiavellism(T1)						0.437**	0.437**
Leader Reward Omission*Machiavellism							0.269**
Total R^2	0.209	0.246	0.346	0.078	0.211	0.328	0.392
Δ R^2	0.191	0.226	0.326	0.057	0.190	0.307	0.370
F	11.821**	12.138**	16.810**	3.788**	9.964	15.459**	17.846**

*p<.05. **p<.01

Supplementary Table 3

Results of Bootstrapping Tests with 95% Confidence Intervals (CI): The Mediating Roles of Moral Disengagement between Leader Reward Omission and Deviant Behavior(Robust test)

Predictor		Effect	SE	*t*	*p*	*LLCI*	*ULCI*
	Total effect	0.148	0.044	3.333	0.001	0.060	0.235
Leader Reward	Direct effect	0.048	0.045	1.077	0.283	-0.040	0.136
Omission		Effect	*Boot S.E.*			*BootLLCI*	*BootULCI*
	Indirect effect	0.099	0.031			0.048	0.171

Supplementary Figure 1

Interaction of Leader Reward Omission and Machiavellianism Predicting Moral Disengagement

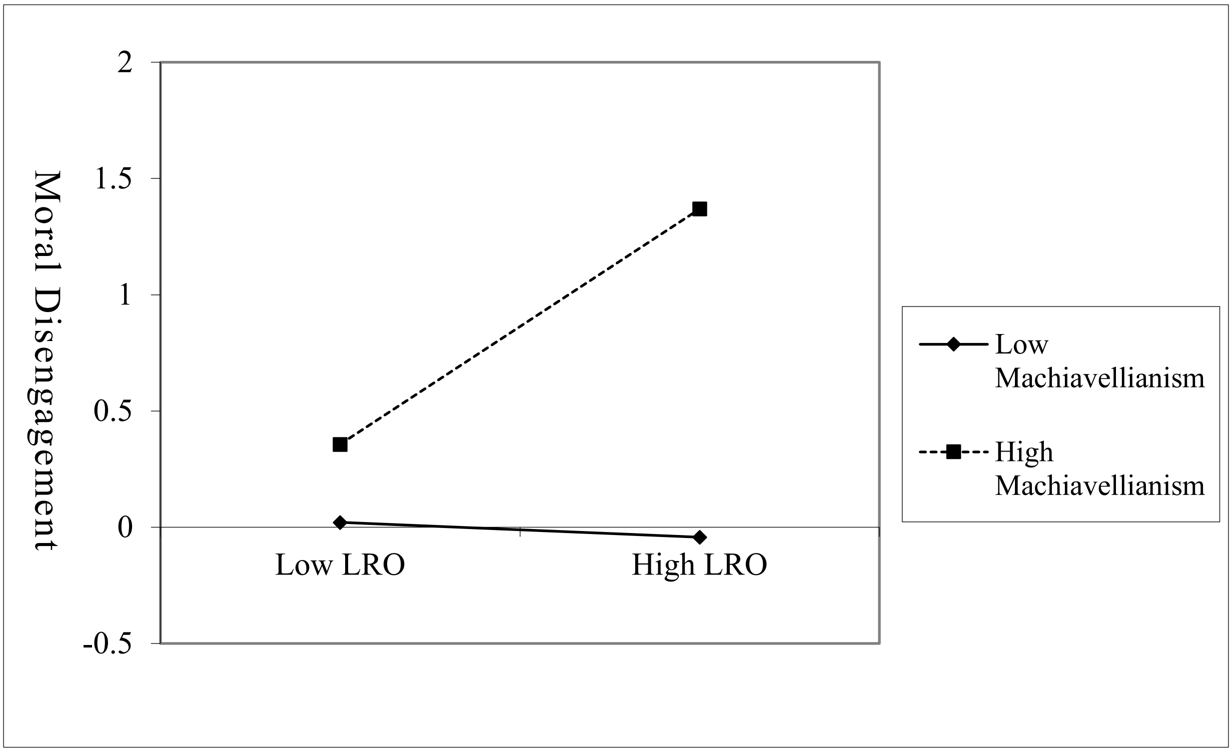

Supplementary Table 4

Results of Bootstrapping Tests with 95% Confidence Intervals (CI): The Moderated Mediation Roles of Machiavellianism between Leader Reward Omission and Deviant Behavior(Robust test)

Predicator	Conditional Indirect Effect					Moderated Mediation			
	Machiavellianism	Effect	Boot SE	BootLLCI	BootULCI	INDEX	SE(Boot)	BootLLCI	BootULCI
leader reward omission	-0.729	-0.004	0.022	-0.042	0.047	0.090	0.025	0.048	0.147
	0.000	0.061	0.023	0.026	0.115				
	0.729	0.126	0.034	0.069	0.205				

REFERENCE

Ferris, D. L., Brown, D. J., Lian, H., & Keeping, L. M. (2009). When does self-esteem relate to deviant behavior? The role of contingencies of self-worth. *J. Appl. Psychol.* 94, 1345–1353. doi:10.1037/a0016115

Ambrose, M. L., & Schminke, M. (1999). Sex differences in business ethics: The importance of perceptions. *J. Manage.Iss.* 11,454–474. https://www.jstor.org/stable/40604285

Gonzalez-Mulé, E., DeGeest, D. S., Kiersch, C. E., & Mount, M. K.(2013). Gender differences in personality predictors of counterproductive behavior. *J. Manage. Psychol.* 28, 333–353. doi:10.1108/JMP-12-2012-0397

Loe, T. W., Ferrell, L., & Mansfield, P. (2000). A review of empirical studies assessing ethical decision-making in business. *J. Bus. Ethics.* 25, 185–204. doi:10.1023/A:1006083612239

Khazanchi, D. (1995). Unethical behavior in information systems: The gender factor. *J. Bus. Ethics.* 14, 741–749.doi:10.1007/BF00872327

Rest, J. R., & Thoma, S. J. (1985). Relation of moral judgment development to formal education. *Dev. Psychol.* 21, 709–714. doi:10.1037/0012-1649.21.4.709
